# Manual reduction of articular disc after traumatic extraction of mandibular third molar: a case report

**DOI:** 10.1590/2177-6709.20.5.101-107.oar

**Published:** 2015

**Authors:** Rubens Camino, Marcello Roberto Manzi, Matheus Furtado de Carvalho, João Gualberto de Cerqueira Luz, Angélica Castro Pimentel, Maria Cristina Zindel Deboni

**Affiliations:** 1 PhD resident in Oral and Maxillofacial Surgery, Universidade de São Paulo (USP), São Paulo, São Paulo, Brazil; 2 Full professor of Oral and Maxillofacial Traumatology, Universidade de São Paulo (USP), School of Dentistry, Department of Oral and Maxillofacial Surgery and Traumatology, São Paulo, São Paulo, Brazil; 3 Professor, Universidade de Santo Amaro (UNISA), Postgraduate Program, São Paulo, São Paulo, Brazil; 4 Associate professor of Oral and Maxillofacial Surgery, Universidade de São Paulo (USP), São Paulo, São Paulo, Brazil

**Keywords:** Temporomandibular joint disc, Magnetic resonance imaging, Symptom assessment

## Abstract

**Introduction:**

Disc displacement without reduction with limited opening is an intracapsular biomechanical disorder involving the condyle-disc complex. With the mouth closed, the disc is in an anterior position in relation to the condylar head and does not reduce with mouth opening. This disorder is associated with persistent limited mandibular opening.

**Case report:**

The patient presented severe limitation to fully open the mouth, interfering in her ability to eat. Clinical examination also revealed maximum assisted jaw opening (passive stretch) with less than 40 mm of maximum interincisal opening. Magnetic resonance imaging was the method of choice to identify the temporomandibular disorders.

**Conclusion:**

By means of reporting this rare case of anterior disc displacement without reduction with limited opening, after traumatic extraction of a mandibular third molar, in which manual reduction of temporomandibular joint articular disc was performed, it was possible to prove that this technique is effective in the prompt restoration of mandibular movements.

## Abstract (pt)

**Introdução:**

o deslocamento do disco articular sem redução com abertura limitada é uma desordem intracapsular que envolve o complexo côndilo-disco. Na posição de boca fechada, o disco articular se encontra numa posição anterior em relação à cabeça da mandíbula e não sofre redução com a abertura de boca. Essa desordem está associada à abertura mandibular limitada e persistente.

**Caso clínico:**

o paciente relatava travamento da mandíbula que não permitia uma abertura completa da boca, interferindo, assim, na capacidade de se alimentar. Também era possível observar-se uma abertura assistida (alongamento passivo) com uma distância vertical menor que 40 mm entre os incisivos. A ressonância magnética foi o método de escolha para o diagnóstico das desordens temporomandibulares.

**Conclusão:**

por meio da descrição de um caso raro de deslocamento anterior do disco articular sem redução e com abertura limitada, após exodontia traumática do terceiro molar inferior, em que foi realizada a redução manual do disco articular da articulação temporomandibular, provou-se ser essa uma técnica eficaz no rápido restabelecimento dos movimentos mandibulares.

## INTRODUCTION

Treatment of temporomandibular joint (TMJ) may be difficult due to the existence of several types of temporomandibular disorders (TMD) with similar clinical signs and symptoms. This complexity forces the professional to master several pathologies of the masticatory system and have clinical experience, so as to achieve differential diagnosis.

TMDs are a significant public health problem affecting about 5 to 12% of the North American population.^1^ In the United States, their prevalence among adults is of 40 to 75% for the presence of at least one sign, and 33% for the presence of at least one symptom. TMDs are commonly found among individuals aged between 20 and 50 years old, and are more frequently present in women than men.^2^

According to Poveda Roda et al,^3^ although there is no defined etiology, several risk factors may be associated with TMD, for instance: age, sex, local or systemic ligamentous laxity, parafunctional habits, trauma, bruxism and stress.

According to the Diagnostic Criteria for Temporomandibular Disorders (DC/TMD),^4^ TMDs are divided into two groups: pain-related disorders and intra-articular temporomandibular disorders. The group of pain-related disorders includes myalgia, local myalgia, myofascial pain, myofascial pain with referral, arthralgia and headache attributed to TMD. As for the group of intra-articular temporomandibular disorders, disc displacement with reduction, disc displacement with intermittent locking, disc displacement without reduction with limited opening, disc displacement without reduction and without limited opening, degenerative joint disease and subluxation are present.

Milano et al^5^ analyzed the prevalence of disc displacement and deformations using magnetic resonance images of symptomatic temporomandibular disorders. Anterior disc displacement with reduction (ADDWR) and anterior disc displacement without reduction (ADDWoR) were the most common types of TMD.

Disc displacement without reduction and with limited opening is an intracapsular biomechanical disorder involving the condyle-disc complex. With the mouth closed, the disc is in anterior position in relation to the condylar head and does not reduce with mouth opening. Medial and lateral displacement of the disc may also be present. This disorder is associated with persistent limited mandibular opening that does not reduce with the clinician or patient performing a manipulative maneuver. This is also referred to as "closed lock".^4^

The criteria for disc displacement without reduction and with limited opening are positive when the patient presents both of the following^4^: jaw locked, so that the mouth would not fully open; and limitation in jaw opening, severe enough to limit jaw opening and interfere in his/her ability to eat. Physical examination reveals maximum assisted opening (passive stretch), including vertical incisal overlap < 40 mm.

TMD patients treatment typically begins with nonsurgical approaches, such as intraoral appliances, dietary modification, physical therapy, medication and occlusal adjustments.^6^^,^ 7 ^,^ 8 ^,^ 9 Eventually, some patients require surgical treatment; but, in general, surgery should only be considered when nonsurgical procedures are not enough to achieve patient's satisfaction on solving joint dysfunction or pain. Some types of surgery have been advocated, such as arthrocentesis, open approach to the articular disc of the TMJ through anchoring, and repositioning of the articular disc.^10^^,^ 11 When facing acute ADDWoR, initial therapy should include the attempt to spontaneously reposition the disc by the patient himself or by means of professional manual manipulation.^12^^,^ 13 ^,^ 14

Thus, the aim of this study is to describe a rare case of anterior disc displacement without reduction and with limited opening after traumatic extraction of a mandibular third molar, treated with the technique of manual reduction of TMJ articular disc.

## CASE REPORT

**Figure 1:**
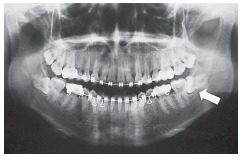
- Panoramic radiograph showing orthodontic indication for extraction of tooth #38.

**Figure 2:**
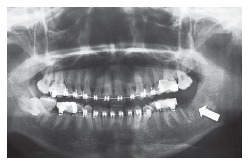
- Panoramic radiograph after extraction of tooth #38, also showing accidental avulsion of teeth #37.

A 23-year-old woman was referred to the Oral and Maxillofacial Surgery Service of Associação Paulista de Cirurgiões-Dentistas 30 days after accidental extraction of a mandibular second molar during extraction of a third molar on the left side. Figures 1 and 2 show the panoramic radiographs taken before and after extractions, respectively. The patient also reported that the surgeon had considerable difficulty during the surgical procedure, which imposed an extensive period of time with the mouth opened. She reported severe joint pain on the right side soon after surgery, accompanied by difficulty in mouth opening and a deviation to the opposite side of the extractions.

**Figure 3:**
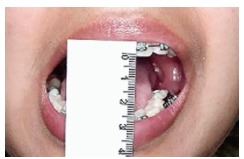
- Mouth opening measuring 23 mm.

**Figure 4:**
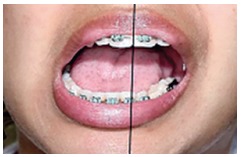
- Mandibular deviation to the right.

**Figure 5:**
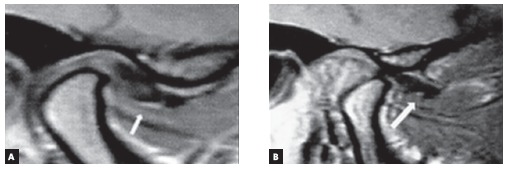
- A) Detail of MRI sagittal slice of the right TMJ with mouth closed, evincing displacement of the TMJ articular disc. B) MRI sagittal slice of the right TMJ at maximal mouth opening, in which ADDWR is evident.

Clinical examination also revealed 23-mm mouth opening (Fig 3), with normal eccentric movement to the ipsilateral side and restriction of eccentric movement to the contralateral side (Fig 4). The following hypotheses were considered: permanent trismus, mandibular fracture or ADDWoR on the right side of the TMJ. Patient denied having history of symptoms related to TMD before surgery. Magnetic resonance imaging (MRI) confirmed ADDWoR on the right side of the TMJ (Figs 5A, B).

The patient was advised to try to reduce deviation by performing lateral movements, as much as possible, to the contralateral side of displacement, and, from this position, try to reach maximum mouth opening. At that time, no increase in mandibular opening and laterality was observed. Thus, two attempts of manual manipulation were performed within the period of one week. In the first attempt, we chose to test mandibular reduction using extraoral anesthesia alone. Due to failure and patient's discomfort, we decided to wait a week before making a new attempt. At this time, we applied the same type of extraoral anesthesia associated with intravenous sedation, thus contributing to successful reduction of disc displacement.

**Figure 6:**
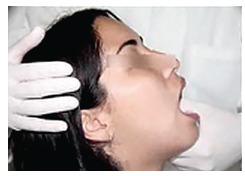
- Manual manipulation for reduction of ADDWoR of TMJ.

Extraoral anesthesia was applied by blocking the auriculotemporal nerve with 1.8 ml of 2% lidocaine hydrochloride associated with norepinephrine 1:200,000, followed by anesthesia of masseteric and posterior deep temporal nerves with the same amount of anesthetics. With a view to providing the patient with greater comfort, an intravenous injection of 2 g midazolam hydrochloride was administered ten minutes before the manual reduction procedure (Fig 4). Thus, 40-mm mouth opening and immediate improvement of mandibular functions were achieved (Fig 6).

Minagi et al's^13^ technique for mandibular manipulation was used. It assists patients in performing maximum lateral excursive jaw movements to the nonaffected side with teeth slightly occluded, and in making maximal jaw opening movements through lateral border movements, as follows: 1) Place the thumb and forefinger on the maxillary canine on the nonaffected side and the mandibular canine on the affected side. Hold the gonion with the forefinger and middle finger of the other hand. 2) Instruct the patient to make maximal lateral gliding excursive jaw movements to the nonaffected side with teeth slightly occluded. Support movement with fingers and ensure that lateral excursive position is maximal. Lateral excursion with the jaw protruding is not adequate for this procedure. 3) Subsequently, instruct the patient to make jaw opening movements through the lateral border path on the nonaffected side. Support this opening movement with assisting fingers. 4) Continue to support voluntary mouth opening up to the maximal opening position.

**Figure 7:**
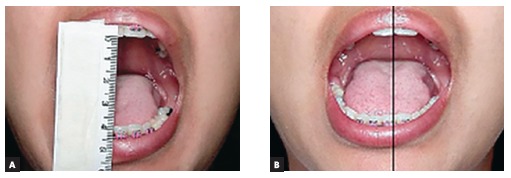
- A) Immediate 40-mm mouth opening after manual manipulation. B) Improvement in mandibular function.

**Figure 8:**
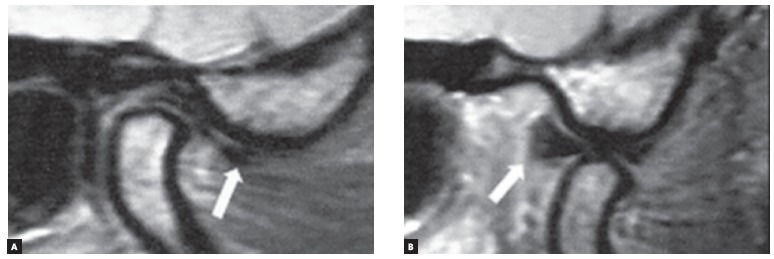
- A) MRI sagittal slice of the right TMJ with the mouth closed, evincing the persistence of TMJ articular disc displacement. B) MRI sagittal slice of the right TMJ at maximal mouth opening, evincing reduction of TMJ articular disc.

The patient received a prescription of anti-inflammatory drugs (100 mg of nimesulide, 12/12 hours, orally) during five days, and also was advised not to force mandibular movements after reduction. The patient was instructed to use a stabilizer plate immediately after correct manipulation, so as to avoid a new disc displacement and reduce muscle hyperactivity. There were no complications after the manipulation maneuver, and an immediate 40-mm mouth opening was achieved after manual manipulation (Figs 7A, B). The patient was followed-up on a weekly basis in the first month and every two weeks until the third month, showing no episodes of TMD within this period. Figures 8A and 8B show MRI sagittal slices of the right TMJ with closed mouth presenting disc displacement and in maximum mouth opening movement, evincing reduction of TMJ articular disc.

## DISCUSSION

Although studies on TMD have been performed for over 70 years, no consensus has been reached regarding its etiology. Lack of a clear single cause conducted a multifactorial etiology. Two hypotheses (occlusal disharmony and psychological distress) have prevailed in the literature.^15^

According to Huang et al,^16^ extraction of third molars seems to be related to TMD. The same authors investigated whether these extractions are a risk factor for TMD in all age groups, and found that the risk was slightly higher in patients under 21 years of age, although difference was not statistically significant.^17^ In the case reported herein, extensive surgery time and the application of considerable forces on the jaw caused trauma on the TMJ and/or masticatory muscles. Thus, we highlight the importance of an improved technique for surgical removal of impacted third molars, so as to avoid keeping the mouth opened for long periods of time. Additionally, intraoral devices are recommended to maintain jaw stability during surgery.

The reason why women are the majority of patients presenting for treatment remains unclear. Although the prevalence of one or more signs of mandibular pain and dysfunction is high in the overall population, only about 5 to 7% have symptoms severe enough to demand treatment.^18^ De Leeuw^2^ reported that after clinical worsening, as the situation becomes chronic, mouth opening tends to increase and the symptoms become milder due to adaptive changes in joint tissues. This situation was not reported by the patient who described that pain got worse day by day.

Several attempts to classify TMD have been made,^19^^,^ 20 ^,^ 21 ^,^ 22 and the division between pain-related disorders and intra-articular temporomandibular disorders is the newest classification. In comparison to the original DC/TMD protocol, the new DC/TMD includes a valid and reliable Axis I screening questionnaire applied to identify TMD-related pain, as well as valid and reliable Axis I diagnostic algorithms for the most common pain-related TMD as part of a comprehensive TMD taxonomic classification structure. Diagnostic criteria for all, but one of the most common intra-articular disorders lack adequate validity for clinical diagnosis, but can be used for screening purposes. Information necessary to fulfill Axis I diagnostic criteria is collected from specific examination protocol together with core self-report instruments that assess pain symptoms involving the jaw, jaw locking, noises and headache. Axis II core assessment instruments assess pain intensity, pain disability, jaw functioning, psychosocial distress, parafunctional behavior, and widespread pain. These changes in the core patient assessment instrument set act as a broad foundation for patient assessment and further research. The new DC/TMD includes important additions, deletions and modifications to the original DC/TMD.^4^

The use of MRI should be the reference, since it allows visualization through multiplanar images with high accuracy, great sensitivity and specificity. It also reveals the position of the TMJ articular disc, the conditions of muscle tissues and disc ligament.^23^ It may also depict joint abnormalities not seen by any other imaging method and, thus, is considered the method of choice to make diagnostic assessment of TMJ status.^24^

TMD treatment has been discussed for decades; however, the consensus is that nonsurgical treatment is effective in most cases. Thus, TMJ surgery would only be indicated for patients with interdental derangements refractory to conservative treatment for at least 6 months.^25^

Occlusal splints play a major role in TMD treatment, as they are a low cost treatment modality with high success rates. The stabilization splint - also known as the Tanner appliance, the Fox appliance, Michigan splint, or centric relation appliance - is widely used in cases of anterior disc displacement without reduction.^7^ In the present case, after manual reduction of articular disc displacement, the patient was compliant with the use of the stabilizer plate, which helped in eliminating pain.

As a result, clinical success was achieved, with evident and immediate reestablishment of mandibular movement extension (40 mm). Subsequently, the patient was advised not to force mandibular movements. However, it is noteworthy that the normalization of joint function does not necessarily imply in recapture of articular disc and may be related to the permanence of the articular disc in initial position or displaced to an even more anterior position. Therefore, it is important to perform new MRI after manual reduction maneuver, as illustrated in the case presented herein. In this case, despite good clinical improvement, the final MRI showed that there was disc displacement with reduction, which did not prevent asymptomatic evolution.

The non-recapture of the articular disc acts as a mechanical barrier, thereby compromising translational movement of the condyle and restricting mouth opening. Additionally, the displaced disc may come to adhere to the articular eminence and permanently limit the translational movement of the condyle.^26^

When normal morphology is present, TMJ articular disc is more likely to return to its normal position. However, when morphology is permanently compromised, it is difficult to keep the disc in position. This is the reason why manual manipulation is only effective in mild conditions. Should the attempts of manual reduction of the articular disc fail, we do not recommend multiple attempts in sequence, as they may worsen patient's signs and symptoms. It is suggested that these maneuvers be made respecting a seven-day interval.

If treatment modalities classified as conservative (medication, physical therapy, stabilizing and repositioning occlusal splints, guidelines) do not achieve successful outcomes, the literature recommends minimally invasive techniques (assisted mandibular manipulation with increased hydrostatic pressure, arthrocentesis) or even invasive techniques (arthroscopy, arthroplasty, arthrotomy).^9^

The technique of mandibular assisted manipulation with increased hydrostatic pressure can also be used in cases of disc displacement with or without reduction in the acute phase (with adherence to the fossa, or to the anterior aspect of the articular tubercle). This technique employs a needle that is usually introduced into the compartment above the disc. The needle is used to insert pressurized saline solution, a local anesthetic, or sodium hyaluronate, in order to release adhesions and dilute local algogenic substances.^27^

Arthrocentesis has the same indication of assisted mandibular manipulation, but has a great advantage of being used in acute and chronic cases. Conventionally, two needles are introduced into the compartment above the disc, inserting a solution - that can be a local anesthetic, Ringer's lactate solution, opioids and sodium hyaluronate - so as to perform joint lavage, dilute local algogenic substances, restore intra-articular normal pressure and assess which substances are present in the synovial fluid.^28^

Arthroscopy is a more invasive technique, performed under anesthesia, and generally involving cannulae, trocars and a small-sized arthroscope containing a camera system connected to a monitor. It can promote lysis of adhesion, washing and manipulation of the head/ articular disc complex, myotomy of muscles, biopsy, removal of bone spicules, injection of sclerosing agents, repositioning and stabilizing the disc, among others.^29^

The arthrotomy procedure can be divided into disc anchoring, disc repositioning discectomy with or without interposition of material, tuberculotomy, condylectomy graft, or complete joint replacement. Disc anchoring has been the most used technique and consists in making a perforation in the posterior-lateral portion of the condyle, so as to have an anchor that will support the disc. It is indicated in cases of disc displacement without reduction, in which conservative clinical therapy or minimally invasive surgical procedures have failed.^30^

Therapeutic success is based on correct diagnosis, professional experience and correct indication of surgical technique. Presently, there is lack of longitudinal studies and randomized clinical trials to compare the effectiveness of each therapeutic modality. Firstly, all types of clinical therapies should be attempted, and if conservative treatment outcome is unfavorable, one can employ more complex invasive treatment modalities.

## CONCLUSION

Manual manipulation maneuver is a very well indicated treatment modality when the history of displacement is recent, proving it to be an effective technique in the early restoration of mandibular movements.
